# Liquid chromatography-tandem mass spectrometry for the quantification of ripretinib and its metabolites DP-5439 in human plasma

**DOI:** 10.3389/fphar.2024.1506931

**Published:** 2025-01-06

**Authors:** Jiahui Lin, Aiting Jiang, Juntao Zheng, Jingjing Wu, Hao Li, Shirong Cai, Yulong He, Xiao Chen, Guoping Zhong, Ke-Jing Tang, Xinhua Zhang, Yanzhe Xia

**Affiliations:** ^1^ Department of Pharmacy, The First Affiliated Hospital, Sun Yat-Sen University, Guangzhou, China; ^2^ School of Pharmaceutical Sciences, Sun Yat-Sen University, Guangzhou, China; ^3^ Department of Gastrointestinal Surgery, The First Affiliated Hospital, Sun Yat-Sen University, Guangzhou, China; ^4^ Institute of Clinical Pharmacology, School of Pharmaceutical Sciences, Sun Yat-Sen University, Guangzhou, China; ^5^ Division of Pulmonary and Critical Care Medicine, The First Affiliated Hospital, Sun Yat-Sen University, Guangzhou, China

**Keywords:** therapeutic drug monitoring (TDM), pharmacokinetics, ripretinib, gastrointestinal stromal tumor, LC-MS/MS

## Abstract

**Background:**

Ripretinib, a broad-spectrum tyrosine kinase inhibitor, has been approved for the treatment of advanced gastrointestinal stromal tumors in adult patients. Clinical studies have shown that higher *in vivo* exposure of ripretinib correlates with improved efficacy, highlighting the potential clinical significance of therapeutic drug monitoring. In this study, a simple and stable liquid chromatography-tandem mass spectrometry (LC-MS/MS) method was attempted to be established and validated for pharmacokinetic studies of ripretinib and its metabolite DP-5439 and therapeutic drug monitoring in human plasma.

**Method:**

Ripretinib and DP-5439 were separated by chromatography using a Thermofisher Hypersil GOLD^TM^ C18 HPLC column. The mobile phase for gradient elution is composed of 0.1% formic acid in water and acetonitrile. Multiple reaction monitoring was implemented along with electrospray ionization positive mode for detection. The ion pairs of ripretinib, DP-5439 and internal standard D8-ripretinib were m/z 510.1→m/z 417, m/z 496.11→m/z 402.9 and m/z 518.15→m/z 420, respectively. Plasma samples from ripretinib-treated patients of our hospital were collected for pharmacokinetic analysis.

**Results:**

Ripretinib and DP-5439 demonstrated a strong linear relationship over 10–5,000 μg/L (*R* ^2^ > 0.99). Accuracy, precision, specificity, recoveries, matrix effect, stability, and dilution effect were all validated and found to meet the required criteria. Following validation, the method was utilized to determine plasma samples from patients treated with ripretinib. The median steady-state trough concentrations (C_min_, range) were 398.50 (66.98 ∼ 1,458.91) μg/L for ripretinib and 654.74 (30.71 ∼ 1,522.48) μg/L for DP-5439, with a total median concentration of 1,129.46 (140.95 ∼ 2,981.39) μg/L in patients receiving ripretinib at 150 mg once daily. Meanwhile, using the established methods, the study conducted pharmacokinetics studies on four patients with ripretinib and DP-5439.

**Conclusion:**

This study developed and validated a robust LC-MS/MS method for determining ripretinib and its metabolite DP-5439 in human plasma. Furthermore, the practicality of this method in clinical sample analysis was demonstrated. This approach can serve as an effective tool for the pharmacokinetics analysis and therapeutic drug monitoring in patients treated with ripretinib.

## 1 Introduction

Gastrointestinal stromal tumor (GIST), commonly occurring in the gastrointestinal tract, is a mesenchymal cell tumor usually driven by activating mutations in the receptor tyrosine kinase proto-oncogene, *KIT*, or the *platelet-derived growth factor receptor α* (*PDGFRα*) (Mehren and Joensuu, 2018; Di Vito et al., 2023; Zalcberg, 2021). Tyrosine kinase inhibitors (TKIs) such as imatinib, sunitinib and regorafenib serve as the first, second, and third line treatment, respectively, by targeting *KIT* and *PDGFRA*. Additionally, avapritinib is specifically utilized for GIST cases harboring *PDGFRA* exon 18 mutations. These therapies have demonstrated promise in extending patient survival and improving outcomes. They do not completely prevent secondary mutations that cause tumor progression over time (Mehren and Joensuu, 2018; Di Vito et al., 2023; Janku et al., 2020; Naito et al., 2023).

By inhibiting kinase signaling of primary and secondary-resistant *KIT* and *PDGFRA* mutations by a dual action mechanism, ripretinib is a broad-spectrum switch-controlled kinase inhibitor that offers a highly favorable safety and efficacy profile in patients with advanced GISTs in clinical studies (Smith et al., 2019; Dhillon, 2020). In the phase III INTRIGUE trial, ripretinib demonstrated comparable efficacy to sunitinib in patients with disease progression or intolerance to imatinib (median progression-free survival (PFS) in *KIT* exon 11 intent-to-treat (ITT) populations, 8.3 versus 7.0 months, respectively, *P* = 0.36), with less adverse events and improved tolerance compared to sunitinib (Bauer et al., 2022). In a phase 2, multicenter, randomized, open-label bridging study of the INTRIGUE study conducted in China, PFS was longer for ripretinib compared to sunitinib in the *KIT* exon 11 ITT population (median PFS not reached for ripretinib versus 4.9 months for sunitinib, *P* = 0.03) (Li et al., 2023). Given its proven efficacy and safety demonstrated in clinical trials, ripretinib has been approved by the National Medical Products Administration of China as well as the U.S. Food and Drug Administration for treating advanced GISTs in adult patients who have undergone three or more kinase inhibitors, including imatinib. Furthermore, ripretinib is also recommended as an alternative second-line therapy in the National Comprehensive Cancer Network (NCCN) Clinical Practice Guidelines in Oncology for Gastrointestinal Mesenchymal Tumors Version 1.2024 and the 2023 edition of Guidelines of Chinese Society of Clinical Oncology (CSCO) for the Diagnosis and Treatment of Gastrointestinal Mesenchymal Tumors (Janku et al., 2020; Smith et al., 2019; Klug et al., 2022; Blay et al., 2020; Bauer et al., 2022; National Comprehensive Cancer Network, 2024; China Clinical Oncology Society Guidelines Working Committee, 2023). In addition, ripretinib demonstrated varying activity in inhibiting different exon locations of *KIT* mutations *in vitro*, which was also observed in the mutational subgroup assessment from the INTRGUE study (Smith et al., 2019; Heinrich et al., 2024). Improved PFS was observed with sunitinib compared to ripretinib in patients with only *KIT* exon 11 + 13/14 mutations (median, 15.0 versus 4.0 months). Conversely, ripretinib showed better PFS versus sunitinib in patients with only *KIT* exon 11 + 17/18 mutations (median, 14.2 versus 1.5 months) (Heinrich et al., 2024). Ongoing research has been conducted continuously in different mutation types to maximize the efficacy and safety of ripretinib for patients with GIST.

Ripreintib is primarily metabolized by N-demethylation, producing the active metabolite DP-5439. The anti-tumor activity of DP-5439 is similar to ripretinib, and both undergo hepatic metabolism primarily via the CYP3A4 enzyme (Li et al., 2022b; Pan et al., 2023). Clinical pharmacokinetic (PK) studies revealed considerable variability in PK parameters among patients, with maximum observed plasma concentration (C_max_) and area under the concentration-time curve from time 0–24 h (AUC_0-24h_) of both ripretinib and DP-5439 increasing proportionally to the dose within a certain dose range, whose variability (coefficient of variation, CV%) amounted to 35 ∼ 60% (Janku et al., 2020). Preclinical studies indicated that the inhibitory effect of ripretinib on *KIT* was both concentration-dependent and time-dependent, suggesting that higher *in vivo* concentrations correlated with increased effectiveness in inhibiting KIT phosphorylation. Additionally, dose escalation had demonstrated improved tumor regression and enhanced survival rates (Smith et al., 2019; Dhillon, 2020). This was confirmed in its phase I clinical study and the INVICTUS phase III trial, where patients initially receiving 150 mg QD orally experienced disease progression (PD) showed renewed benefit after dose escalation to 150 mg BID (Blay et al., 2020; Janku et al., 2020). This suggested that increased exposure of ripretinib correlated with improved efficacy (George et al., 2021; Zalcberg et al., 2021). Furthermore, considering the differences in ripretinib effects across various mutations observed in in vitro and *in vivo* studies, it can be speculated that the exposure to ripretinib may vary among these mutational subgroups, warranting further investigation.

In the phase I dose-escalation trial for ripretinib, no maximum tolerated dose (MTD) was identified. However, dose-limiting toxicities (DLTs) such as elevated lipase and creatine kinase were observed in the 100 mg BID, 200 mg BID, and 150 mg QD dose groups. Adverse events including myalgia, muscle spasms, palmar-plantar erythrodysesthesia (PPES), and hypertension exhibited a dose-dependent increase, indicating potential dose-escalation-related toxicity with ripretinib (Janku et al., 2020). This highlights the importance of exploring ripretinib exposure to mitigate adverse events. Consequently, therapeutic drug monitoring (TDM) of ripretinib could be clinically significant for patients. Moreover, further investigation into the pharmacokinetic and pharmacodynamic properties of ripretinib is essential to enhance patient outcomes and facilitate its clinical utility.

Several quantification methods for ripretinib in beagle dogs and rats plasmas have been developed. An ultra-performance liquid chromatography-tandem mass spectrometry method (UPLC-MS/MS) was established by Wang et al. (2021) to measure ripretinib levels in beagle dogs, and the impact of voriconazole and itraconazole on the PK of ripretinib was investigated. Similarly, Mudavath and Ashok (2023) developed a LC-MS/MS method to quantify ripretinib in rat plasma. However, few methods remain for monitoring blood concentrations of ripretnib and DP-5439 in human plasma. Li et al. (2022b) reported the effect of CYP3A initiative, CYP3A inspiration, and gastric acid reduction on the pharmacokinetics of ripretinib without detailed quantification methods. Qian et al. (2024) established an LC-MS/MS method for determining blood concentration in human plasma ripretnib and DP-5439. Therefore, the purpose of this study was to develop and validate an LC-MS/MS method for quantifying ripretinib and DP-5439 in human plasma. Subsequently, this method was then applied to clinical samples to assess its practical applicability in GIST patients.

## 2 Materials and methods

### 2.1 Chemicals and reagents

Ripretinib (99.72% purity) was purchased from Target Molecule Corp. (TargetMol) company (Shanghai, Topscience). DP-5439, the metabolite of ripretinib, and D8-ripretinib, the internal standard (IS), were obtained from WuXi App Tec (Nantong) Co., Ltd with a purity > 99%. Acetonitrile, methanol (HPLC grade), and formic acid (FA) (LC/MS grade) were obtained from Thermo Fisher Scientific Inc., China. DMSO (BioReagent) was obtained from Sigma-Aldrich Co LLC. Ultrapure water was produced by Thermo Scientific™ Barnstead™ MicroPure™ water purification system.

### 2.2 Instrumentation

An Agilent 1260 HPLC was employed in the LC-MS/MS analysis, consisting of a G1312B binary pump, Hip Sampler (G1367E) Autosampler, and Column Comp. (G1316A), linking to a 6420 triple-quadrupole mass spectrometer with an electrospray ionization source (ESI) (Agilent, CA, United States). Original data were acquired using Agilent MassHunter data acquisition software (version B.07) and analyzed with Quantitative analysis software (version B.07). Other instruments: Vortex mixer xw-80A (Haimen Qilinbel Instrument Manufacturing Co., Ltd.); Benchtop High-Speed Centrifuge Jiawen JW-3021H (Anhui Jiawen Instrument Equipment Co., Ltd.); Ultra-low Temperature and High-Speed Centrifuge 5810R (Eppendorf AG 22331 Hamburg Germany); Thermo Scientific™ Barnstead™ MicroPure™ Ultrapure Water Meter (Item No. 50132373, Thermo Electron LED GmbH).

### 2.3 Chromatography and mass spectrometry

The analyte was separated using a Thermofisher Hypersil GOLDTM C18 HPLC column (4.6 mm × 50 mm, 5 μm) at a column temperature of 35°C. The analysis time was 6.0 min, and the mobile phase flow rate was set to 0.5 mL/min. For gradient elution, 0.1% (v/v) formic acid in water was chosen as mobile phase A, and acetonitrile as mobile phase B. The procedure is configured as follows: 0 min ∼ 1 min, 20% B; 3 min ∼ 5.00 min, 90% B; 5.01 ∼ 6 min, 20% B. The injection volume was 5 μL.

**FIGURE 1:**
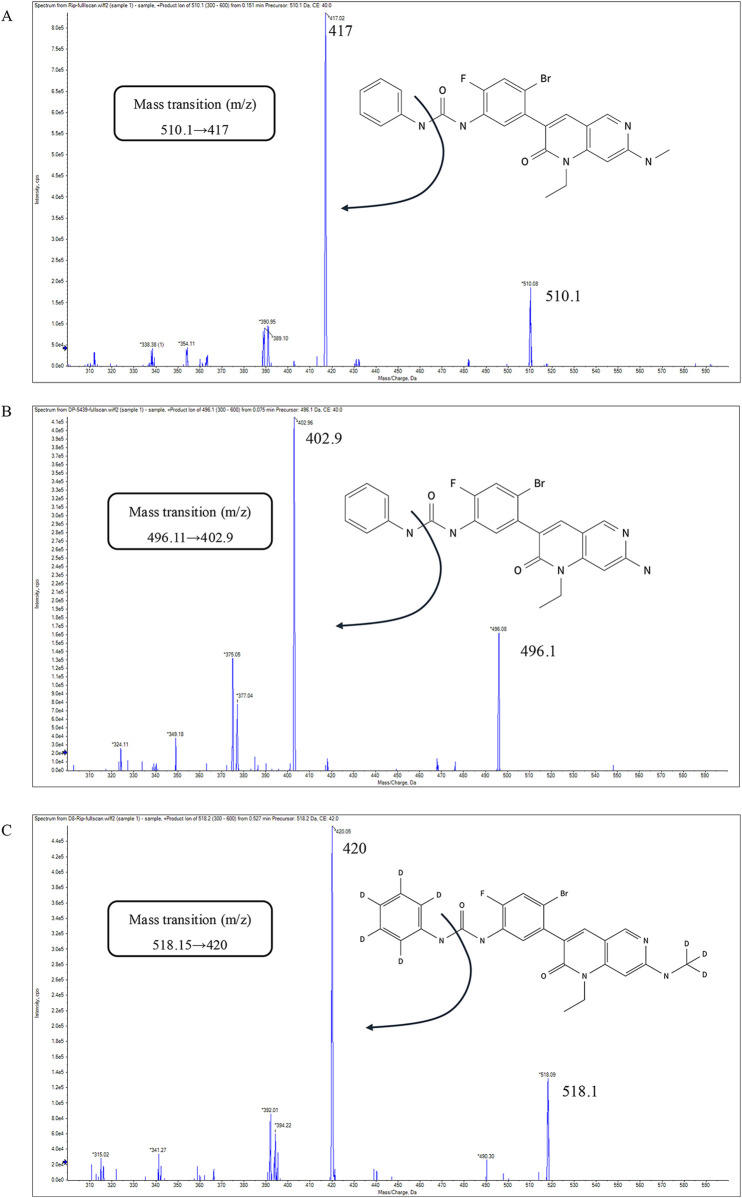
The chemical structure, fragmentations and the product-ion mass spectra of ripretinib **(A)**, DP-5439 **(B)**, D8-ripretinib **(C)**.

Positive electrospray ionization (ESI+) in multiple reaction monitoring (MRM) mode was employed for the mass spectrometry procedure. The retention times for ripretinib, DP-5439, and D8-ripretinib were 4.11, 3.99, and 4.09 min, respectively. The mass transition, fragmentor, and collision energy details were provided in Table 1. The chemical structure and fragmentations of ripretinib, DP-5439, and D8-ripretinib were illustrated in Figure 1. The ion source settings were: 4.0 kV for capillary voltage, 350°C for gas temperature, 10 L/min for gas flow rate, and 40 psi for nebulizer pressure.

**TABLE 1:** Monitored transitions, fragmentor and collision energy of ripretinib, DP-5439 and D8-ripretinib.

Analyte	Mass transition (m/z)	Dwell	Fragmentor	Collision Energy, V
Ripretinib	510.1→ 417	200	230	34
DP-5439	496.11→ 402.9	200	210	30
D8-Ripretinib	518.15→ 420	200	230	34

### 2.4 Standard solutions

DMSO was used as a solvent to prepare stock standard solutions of ripretinib (10 mg/mL). The same method was used to prepare the stock standard solutions of DP-5439 (10 mg/mL) and D8-ripretinib (10 mg/mL). Then the stock solutions of ripretinib and DP-5439 were diluted to 1 mg/mL using DMSO. The working standard solutions (ripretinib, 0.2, 0.5, 1, 2, 5, 10, 20, 50, 100 μg/mL and DP-5439, 0.2, 0.5, 1, 2, 5, 10, 20, 50, 100 μg/mL), working quality control (QC) solutions (ripretinib, 0.2, 0.6, 6, 20, 75 μg/mL and DP-5439, 0.2, 0.6, 6, 20, 75 μg/mL) and the IS solution (D8-ripretinib, 2.0 μg/mL) were prepared by gradient dilution with 85% methanol. All the stock and working standard solutions were stored at 4°C.

### 2.5 Sample preparation

The samples were prepared as follows: 100 μL of plasma was mixed with 5 μL of IS solution and then precipitated with 300 μL of acetonitrile. The mixture was centrifuged at 12,000 g for 8 min at 4°C after an intense vortex for 1 min. Subsequently, 200 μL of supernatant was moved into LC-MS vials, and 5 μL was utilized for analysis with LC-MS/MS.

The calibration standard samples and the QC samples were prepared by mixing human plasma with the working standard solutions and the QC solutions. The calibration standard points were 10, 25, 50, 100, 250, 500, 1,000, 2,500 and 5,000 μg/L for both ripretinib and DP-5439. Lower limit of quantitation (LLOQ), low QC (LQC), medium QC (MQC), sub-high QC (SHQC), and high QC (HQC) points were 10, 30, 300, 1,000, 3,750 μg/L both for ripretinib and DP-5439.

### 2.6 Assay validation procedures

The method validation was conducted under *9012 Guidelines for Validation of Biological Sample Quantitative Analysis Methods* of *Chinese Pharmacopoeia*, as well as *the International Conference on Harmonisation (ICH) guideline M10 “Bioanalysis Method Verification and Sample Analysis”* (Chinese Pharmacopoeia Commission, 2020; International Conference on Harmonization of Technical Requirements for Registration of Pharmaceuticals for Human Use (ICH), 2022), consisting of specificity, linearity, lower limit of quantification, accuracy, precision, matrix effect, extraction recovery, carry over, stability, and dilution effects.

#### 2.6.1 Specificity and selectivity

Blank plasma matrices of 6 distinct individuals were taken to examine the method’s specificity in distinguishing ripretinib, DP-5439, and the IS from all other substances. Observe the presence of interference peaks at the peaks of ripretinib, DP-5439, and the IS in the blank plasma samples, and compare the response of the interferences with the response of ripretinib, DP-5439, and the IS in the LLOQ samples. It is acceptable for the interference peak area in the blank sample to be less than 20% of the peak of ripretinib and DP-5439 at LLOQ and 5% of the IS peak.

#### 2.6.2 Linearity and lower limit of quantification

The standard samples were prepared according to the above “2.5”, each batch includes a set of 9 standard calibrators of known concentration, a blank sample with internal standard and a blank sample without analyte and internal standard. The regression equation was calculated by using the peak area ratio of ripretinib and DP-5439 to the IS and the nominal analyte concentration. The linearity of the standard curves of both analytes was tested by weighting (1/x^2^ weighted coefficient) based on the least-square method. Linearity is considered satisfactory if the correlation coefficient (*r*) surpasses 0.99. The measured concentrations of ripretinib and DP-5439 must fall within a range of ±15% of the expected value, while LLOQ should be within a range of ±20%.

#### 2.6.3 Accuracy and precision

The accuracy and precision of five concentrations were examined: LLOQ, LQC, MQC, SHQC, and HQC. For intra-day and inter-day accuracy and precision, six replicates of each concentration were measured in parallel within the same batch and three consecutive batches were repeated over a minimum of 2 days. Relative standard deviation (RSD%) represented accuracy, while relative error (RE%) represented precision. The accuracy of the LLOQ samples should not exceed ±20% of the theoretical value, and ±15% for samples of other concentration points.

#### 2.6.4 Matrix effect and extraction recovery

Concentrations at LLOQ, LQC, MQC, SHQC, and HQC were investigated for matrix effects and extraction recovery with matrices from 6 different individuals. The validation was performed in 3 groups as follows: For group 1, blank plasma samples were spiked with analytes and then treated according to “Section 2.5,” which obtained peak area *A*_*1*_. For group 2, ultrapure water was used to replace the blank plasma, then processed in the same way to obtain peak area *A*_*2*_. For group 3, blank plasma from the same sources was taken and pretreated with 300 μL of acetonitrile precipitation before adding the corresponding concentrations of analytes, which were treated to obtain the peak area *A*_*3*_. Matrix factor = *A*_*1*_/*A*_*2*_ × 100%. Extraction recovery = *A*_*1*_/*A*_*3*_ × 100%. The IS-normalized matrix factors for ripretinib and DP-5439 were calculated by dividing their matrix factors by the IS matrix factor, respectively. The CV% of the IS-normalized matrix factor determined from 6 batches of matrix should not exceed 15%. Additionally, the RSD% of the extraction recovery should not exceed 15%.

#### 2.6.5 Carry over

The residual effect was evaluated after the injection of the sample with the highest concentration. The residual peak area of the subsequent blank sample should be less than 20% of the analyte peak for LLOQ and 5% for IS.

#### 2.6.6 Stability

To validate the stability of the prepared samples under various storage conditions, the samples were analyzed after being stored at room temperature for 24 h, autosampler for 24 h, 3 freeze-thaw cycles at −20°C and −80°C, and long-term storage at −20°C and −80°C for 30 days, respectively. Five sets of plasma samples with LLOQ, LQC, MQC, SHQC, and HQC were produced and measured according to the corresponding conditions to investigate stability with a criterion of instability not exceeding ±15%.

#### 2.6.7 Dilution integrity

To evaluate dilution reliability, control samples at 7,500 μg/L (higher than ULOQ) were prepared using blank human plasma. These samples were then diluted 2, 10, 30, and 300 times with blank plasma to obtain concentrations of 3,750, 750, 250, and 25 μg/L. Each dilution factor was tested in parallel on the same batch. The dilution samples should have an average accuracy within a range of ±15% of the labeled value, and the precision (CV%) should be no more than ±15%.

### 2.7 Clinical application

To validate the method’s applicability, plasma samples were collected from GIST patients treated with ripretnib at steady-state trough concentrations. Following a minimum of 15 consecutive days of ripretinib treatment, samples were obtained before the subsequent ripretinib dose and within 24 ± 2 h after the previous dose. Meanwhile, a whole-point pharmacokinetic curve was collected from four patients after reaching steady-state concentrations to study the pharmacokinetics of ripretinib and DP-5439. Blood samples were collected at eight time points: 0 h before administration, and 0.5, 1, 2, 4, 6, 12, and 24 h after administration. After collecting blood samples in EDTA anticoagulant tubes, the plasma was preserved at −20°C following centrifugation. The study received approval from the IEC for Clinical Research and Animal Trials of the First Affiliated Hospital of Sun Yat-sen University (No. [2024]162-1).

### 2.8 Statistical methods

Microsoft Excel, IBM SPSS 26.0 and GraphPad Prism 10.0 were employed to execute data statistical analysis. Non-compartmental pharmacokinetic analysis was performed using Phoenix Certara 8.1.

## 3 Results

### 3.1 Method validation

#### 3.1.1 Specificity and selectivity

**FIGURE 2:**
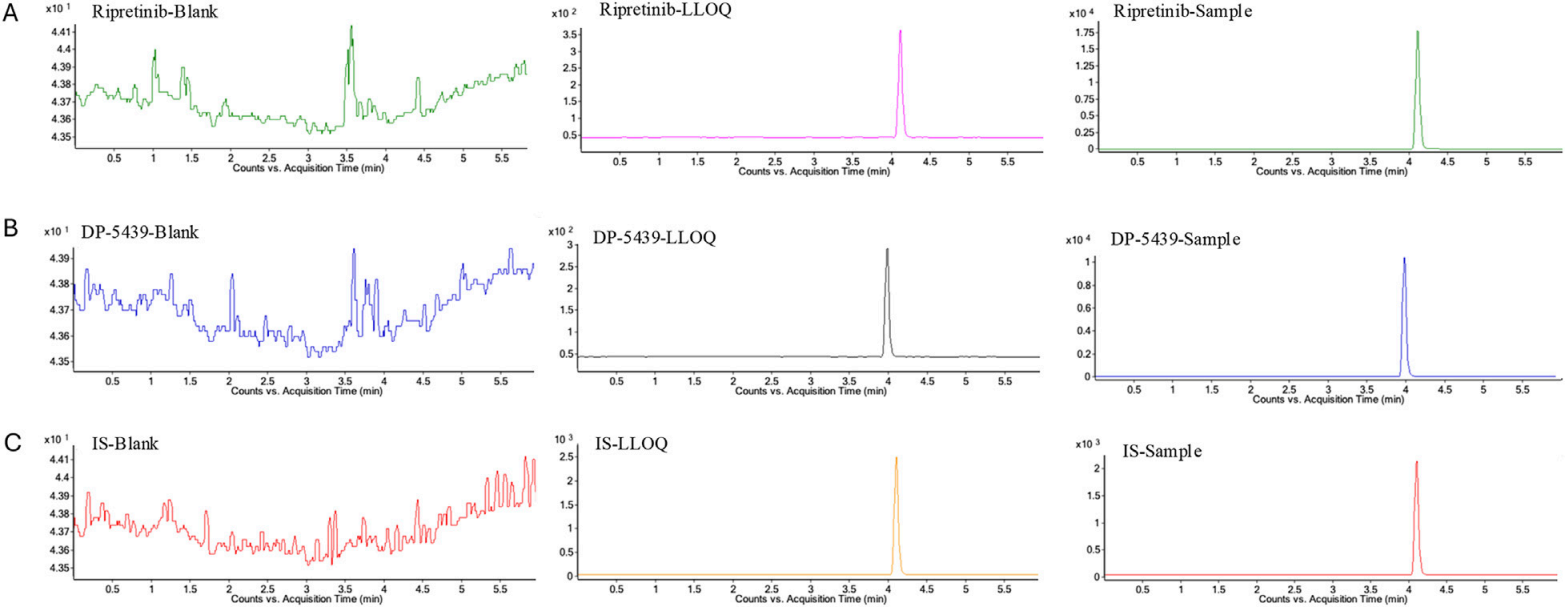
Representative multiple reaction monitoring chromatograms of ripretinib **(A)**, DP-5439 **(B)**, and IS **(C)** in human plasma. LLOQ: LLOQ sample; Sample: patient plasma sample received ripretinib.

No significant interference was observed at the retention times of ripretinib, DP-5439, or IS in the six distinct blank matrices that were investigated. Meanwhile, the compounds ripretnib, DP-5439, and IS were well separated, with intact peak shapes observed in both clinical and LLOQ samples (Figure 2). This indicates that the method is sufficiently selective and specific.

#### 3.1.2 Linearity and lower limit of quantification

The calibration curve demonstrated satisfactory linearity in the 10–5,000 μg/L range for both ripretinib (*y* = 6.03670*x*-0.011993, *R* ^2^ = 0.9989) and DP-5439 (*y* = 4.173934*x*-0.001953, *R* ^2^ = 0.9981) for DP-5439 (Supplementary Figure S1). The observed concentrations of ripretinib and DP-5439 were within 94.01%∼105.52% of the expected concentrations. Additionally, the accuracy and precision of LLOQ met the requirements of within ±20%.

#### 3.1.3 Accuracy and precision

The intra-day and inter-day accuracy of ripretinib for LLOQ were −2.51% and 3.79%, and 1.12% and 3.63% for DP-5439, with precision below 5.75%. For all other concentrations, the intra-day and inter-day accuracy of ripretinib is between −3.71% and 4.72%, with precision all lower than 4.98%. For LQC, MQC, SHQC, and HQC, the intra-day and inter-day precision of DP-5439 were less than 6.70%, with accuracy ranging from −3.81% to 8.93% (Table 2). The above results demonstrated that the accuracy and precision of this method were sufficient.

**TABLE 2:** Accuracy, precision, matrix effect and recovery of ripretinib and DP-5439.

Analyte	Measurement level	Nominal Conc. μg/L	Intra-day (*n* = 6)				Inter-day (*n* = 18)				Matrix effect (*n* = 6)			Extraction recovery (*n* = 6)		
			Measured conc. (μg/L)	SD	RSD (%)	RE (%)	Measured conc. (μg/L)	SD	RSD (%)	RE (%)	IS-Normalized matrix factor	SD	RSD%	Recoveries in %	SD	RSD%
Ripretinib	LLOQ	10	9.75	0.20	2.05%	−2.51%	10.38	0.60	5.75%	3.79%	94.31%	1.70%	1.80%	93.62%	1.69%	1.80%
	LQC	30	30.43	0.52	1.72%	1.43%	29.41	1.37	4.66%	−1.96%	95.39%	1.22%	1.27%	92.81%	1.18%	1.27%
	MQC	300	293.57	3.41	1.16%	−2.14%	288.86	8.71	3.02%	−3.71%	97.68%	3.07%	3.14%	96.21%	3.02%	3.14%
	SHQC	1,000	1,005.16	11.96	1.19%	0.52%	970.34	44.11	4.55%	−2.97%	98.32%	2.20%	2.24%	95.59%	2.14%	2.24%
	HQC	3,750	3,927.18	65.37	1.66%	4.72%	3,704.02	184.58	4.98%	−1.23%	95.31%	2.85%	2.99%	100.28%	2.99%	2.99%
DP-5439	LLOQ	10	10.11	0.20	1.99%	1.12%	10.36	0.59	5.70%	3.63%	99.29%	9.53%	9.60%	95.41%	9.16%	9.60%
	LQC	30	32.68	1.02	3.11%	8.93%	30.67	2.05	6.70%	2.25%	100.93%	3.46%	3.43%	92.92%	3.18%	3.43%
	MQC	300	305.44	8.53	2.79%	1.81%	295.50	12.58	4.26%	−1.50%	99.63%	3.83%	3.85%	93.46%	3.59%	3.85%
	SHQC	1,000	1,003.50	15.39	1.53%	0.35%	961.94	49.31	5.13%	−3.81%	98.16%	1.84%	1.88%	93.00%	1.75%	1.88%
	HQC	3,750	3,856.02	76.29	1.98%	2.83%	3,637.17	178.98	4.92%	−3.01%	104.22%	2.63%	2.52%	98.61%	2.49%	2.52%

RSD, relative standard deviation; RE, relative error; SD, standard deviation; LLOQ, lower limit of quantification; LQC, low QC; MQC, medium QC; SHQC, sub-high QC; HQC, high QC; QC, quality control.

#### 3.1.4 Matrix effect and recovery

The plasma internal standard normalized matrix effectors ranged from 94.31% to 98.32% for ripretinib and from 99.29% to 104.22% for DP-5439, with RSD less than 15.0% at all concentrations (Table 2), suggesting that the matrix effect was acceptable. The extraction recovery of ripretinib and DP-5439 in plasma samples ranged from 93.62% to 100.28% and 92.92%–98.61%, respectively, with RSD ≤9.60%.

#### 3.1.5 Carry over

There was no significant residue in the blank plasma sample immediately following the analysis of the sample with the highest concentration (Supplementary Table S1). The residual peak area of ripretinib and DP-5439 was less than 20% of LLOQ and below 5% of IS, fulfilling the necessary criteria.

#### 3.1.6 Stability

All stability results tested under different conditions all met the guidelines (Table 3). The sample stability ranged from 88.78% to 106.99%, with RSD less than 15.0%. This indicated that ripretinib and DP-5439 remained stable in human plasma under the following conditions: at room temperature for 24 h, autosampler for 24 h, 3 freeze-thaw cycles at −20°C and −80°C, and long-term storage at −20°C and −80°C for 30 days.

**TABLE 3:** Stability of ripretinib and DP-5439 under various storage conditions (*n* = 6).

Analyte	Measurement level	Room temp. for 24 h		Freeze-Thaw Cycles at −20°C		Freeze-Thaw Cycles at −80°C		Autosampler for 24 h		−20°C for 30 days		−80°C for 30 days	
		Measured conc. (μg/L) (Mean ± SD)	Stability%	Measured conc. (μg/L) (Mean ± SD)	Stability%	Measured conc. (μg/L) (Mean ± SD)	Stability%	Measured conc. (μg/L) (Mean ± SD)	Stability%	Measured conc. (μg/L) (Mean ± SD)	Stability%	Measured conc. (μg/L) (Mean ± SD)	Stability%
ripretinib	LLOQ	9.62 ± 0.17	96.18	9.76 ± 0.24	97.63	9.54 ± 0.37	95.39	10.45 ± 0.25	104.48	10.10 ± 0.27	101.00	10.07 ± 0.26	100.72
	LQC	30.00 ± 1.49	100.01	28.80 ± 1.06	96.00	29.20 ± 0.91	97.33	29.23 ± 0.97	97.44	30.44 ± 0.84	101.46	30.39 ± 0.58	101.28
	MQC	282.86 ± 9.49	94.29	274.81 ± 5.89	91.60	266.34 ± 10.50	88.78	290.03 ± 4.67	96.68	286.96 ± 7.03	95.65	282.30 ± 8.39	94.10
	SHQC	996.50 ± 54.31	99.65	941.12 ± 34.15	94.11	927.93 ± 42.75	92.79	963.94 ± 31.12	96.39	987.13 ± 32.01	98.71	974.62 ± 40.79	97.46
	HQC	3,729.00 ± 160.49	99.44	3,547.57 ± 139.38	94.60	3,596.24 ± 166.78	95.90	3,626.61 ± 56.92	96.71	3,753.00 ± 187.52	100.08	3,902.89 ± 77.66	104.08
DP-5439	LLOQ	10.55 ± 0.30	105.54	10.61 ± 0.46	106.14	10.12 ± 0.43	101.16	9.85 ± 0.47	98.53	9.52 ± 0.44	95.16	9.77 ± 0.48	97.68
	LQC	32.10 ± 1.69	106.99	30.88 ± 1.07	102.92	31.84 ± 1.36	106.15	29.43 ± 1.95	98.11	31.08 ± 1.22	103.58	31.36 ± 1.17	104.55
	MQC	290.82 ± 11.18	96.94	288.90 ± 5.56	96.30	280.29 ± 11.60	93.43	293.38 ± 7.17	97.79	289.84 ± 11.03	96.61	288.97 ± 9.00	96.32
	SHQC	980.95 ± 51.52	98.09	940.32 ± 47.99	94.03	925.65 ± 43.30	92.56	955.00 ± 34.67	95.50	959.56 ± 32.56	95.96	953.95 ± 42.36	95.40
	HQC	3,699.20 ± 171.68	98.65	3,508.90 ± 163.88	93.57	3,569.08 ± 153.77	95.18	3,529.91 ± 58.98	94.13	3,622.55 ± 171.96	96.60	3,772.76 ± 82.24	100.61

RSD, relative standard deviation; SD, standard deviation; LLOQ, lower limit of quantification; LQC, low QC; MQC, medium QC; SHQC, sub-high QC; HQC, high QC; QC, quality control.

#### 3.1.7 Dilution integrity

The diluted QC samples exhibited an imprecision of less than 3.95% with inaccuracy between −7.23% and 6.38% (Supplementary Table S2). It indicated that all the tested dilution factors were practical for samples with concentrations beyond the ULOQ.

### 3.2 Clinical application

**FIGURE 3:**
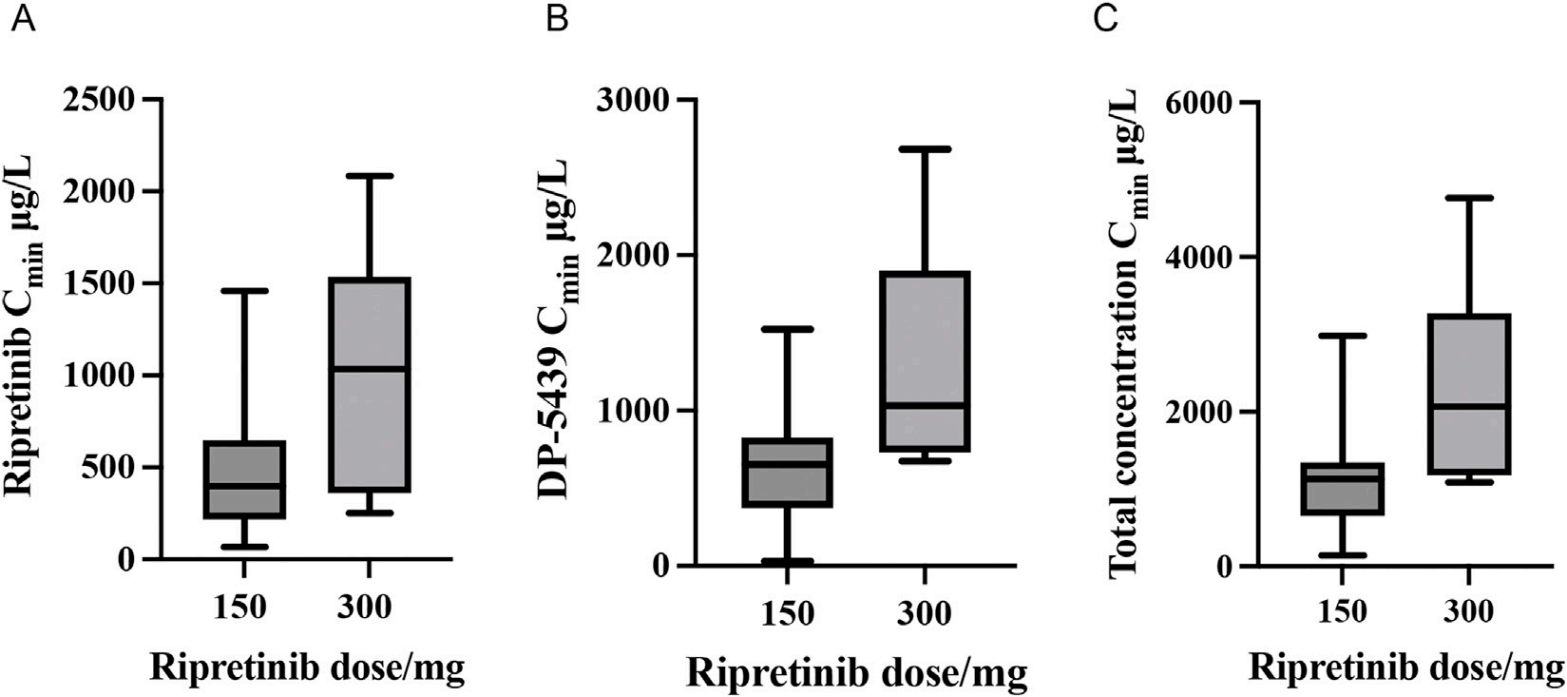
Steady-state trough concentrations (C_min_) of ripretinib and DP-5439 in patients with GIST (**(A)** Ripretinib concentrations; **(B)** DP-5439 concentrations; **(C)** Total concentrations).

A total of 53 plasma samples from 33 patients who received ripretinib were collected to measure steady-state trough concentrations (C_min_). Baseline and demographic characteristics are shown in Table 4. Among these patients, 26 were administered a 150 mg once-daily (QD), 4 received a 150 mg twice-daily (BID) dose, 2 were prescribed a 100 mg, and another initially received 150 mg QD, subsequently escalating to 150 mg BID. At 150 mg QD, the total median C_min_ (range) was 1,129.46 (140.95 ∼ 2,981.39) μg/L, with ripretinib median C_min_ (range) at 398.50 (66.98 ∼ 1,458.91) μg/L and DP-5439 median C_min_ at 654.74 (30.71 ∼ 1,522.48) μg/L. In the 300 mg dose group, the median C_min_ was 1,034.87 (251.36 ∼ 2083.52) μg/L for ripretinib and 1,032.73 (675.27 ∼ 2,682.57) μg/L for DP-5439, resulting in a total median C_min_ was 2067.60 (1,089.64 ∼ 4,766.08) μg/L (Figure 3). Patients received the 100 mg QD dose exhibited median C_min_ of 328.20 (309.43 ∼ 346.96) μg/L for ripretinib and 446.86 (413.58 ∼ 480.14) μg/L for DP-5439.

**TABLE 4:** The characteristics of the patients received ripretinib.

Parameters	*n* = 33
Age (years)	
Median (range)	58 (36–75)
Gender, *n* (%)	
Male	19 (57.6)
Female	14 (42.4)
Number of prior TKIs, *n* (%)	
1	5 (15.2)
2	7 (21.2)
3	14 (42.4)
≥4	7 (21.2)
Primary tumor site, *n* (%)	
Stomach	9 (27.3)
Small intestine	21 (63.6)
Rectum	1 (3.0)
Other sites	2 (6.0)
Metastasis site, *n* (%)	
Liver	25 (75.8)
Peritoneum	30 (90.9)
Bone	3 (0.09)
Lung	2 (0.06)
Primary tumor mutation, *n* (%)	
*KIT* 9	4 (12.1)
*KIT* 11	27 (81.8)
*KIT* 13	1 (0.03)
*KIT/PDGFRα* Wild type	1 (0.03)
Ripretinib dose (*mg/d*)	
100	2
150	26
300	4
150 → 300	1

Blood samples from four patients provided the pharmacokinetic parameter of ripretinib and DP-5439. Three of the patients, patients 1, 2, and 3, had advanced GIST originating from the small intestine with a primary mutation of *KIT* 11 and were treated with ripretinib 150 mg QD as a second-line treatment (Table 5). Patient 4 was a woman with a GIST from the small intestine with metastasis to the liver and peritoneum, treated with ripretinib 150 mg bid as a 5-line treatment. Her gene mutation type was the *KIT* 11 + *KIT* 17 mutation.

**TABLE 5:** The characteristics of four patients who provided pharmacokinetic parameters.

Parameters	Patient 1	Patient 2	Patient 3	Patient 4
Age (years)	60	66	30	44
Gender	Male	Female	Male	Female
Number of prior TKIs	1	1	1	4
Primary tumor site	Small intestine	Small intestine	Small intestine	Small intestine
Recurrence/metastasis site	Liver	Peritoneum	Small intestine	Liver and Peritoneum
Tumor mutation	*KIT* 11	*KIT* 11	*KIT* 11	*KIT* 11+ *KIT* 17
Ripretinib dosage	150 mg *qd*	150 mg *qd*	150 mg *qd*	150 mg *bid*

**FIGURE 4:**
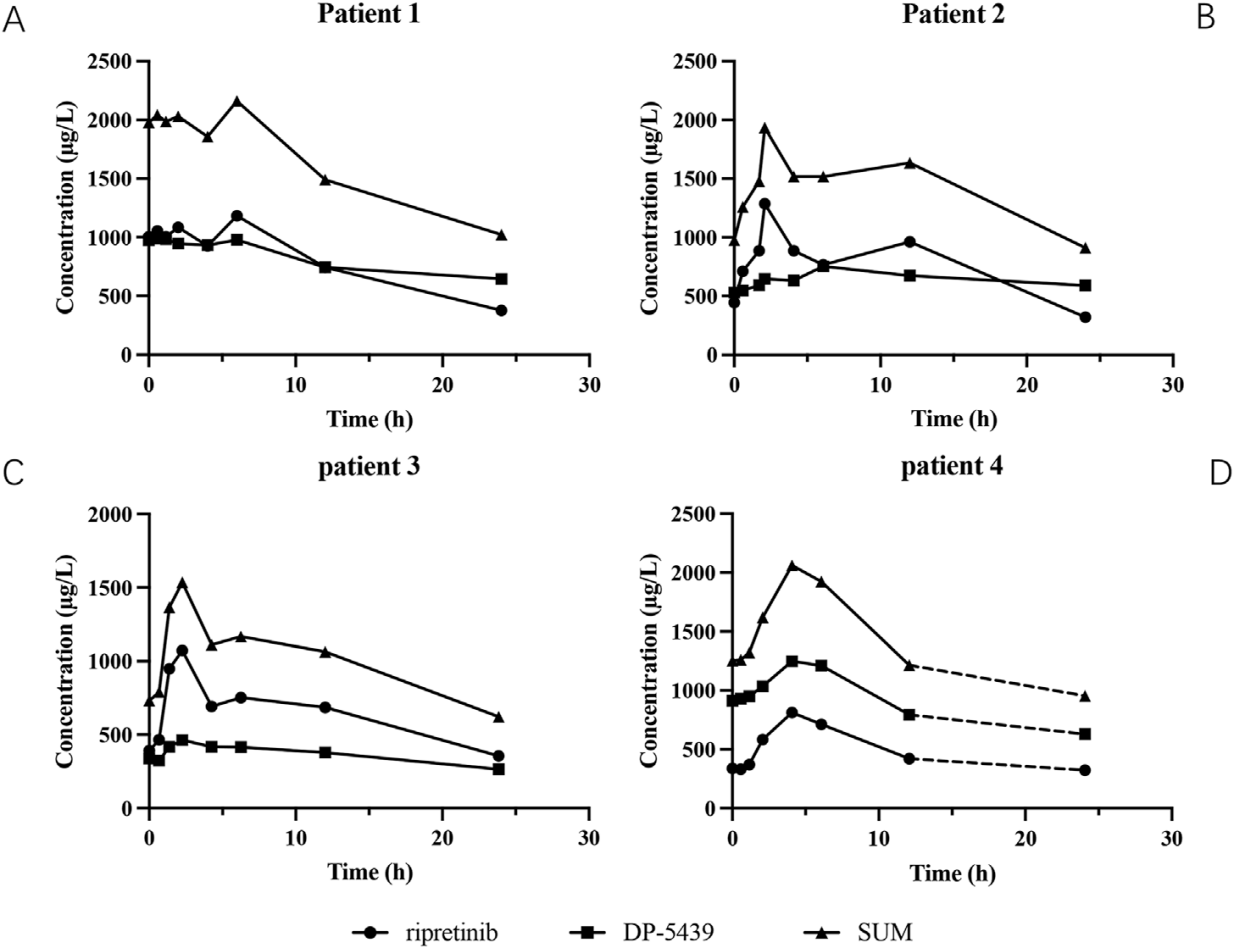
The plasma concentration-time curves of ripreitnib, DP-5439 and total concentration after oral administration. **(A)** Patient 1. **(B)** Patient 2. **(C)** Patient 3. **(D)** Patient 4. The dashed line indicated that the patient was given another 150 mg of ripretinib after 12 h of the first administration and then obtained the concentration 24 h after the first dosage. All patients reached trough concentrations (C_min_) of ripretinib and DP-5439 approximately 24 h after a single dose, while the pharmacokinetic behavior of ripretinib and DP-5439 varied considerably between patients. Time to maximum plasma concentration (T_max_) ranged from 2.07 to 6 h for ripretinib and 0.57–6.07 h for DP-5439. The terminal half-life (t_1/2_) of ripretinib varied from 7.94 to 15.58 h and 9.84–62.95 h for DP-5439. The volume of distribution (V_d_) ranged from 0.22 to 243.17 L for ripretinib and 0.67–403.34 L for DP-5439.

The main pharmacokinetic parameters of the four patients are shown in Table 6, and the plasma concentration-time curves are shown in Figure 4. All patients reached C_min_ of ripretinib and DP-5439 approximately 24 h after a single dose. For patient 1, the T_max_ were 6.00 h for ripretinib and 0.57 h for DP-5439. The C_max_ for ripretinib and DP-5439 were 1,181.88 ng/mL and 989.28 ng/mL, respectively. However, for patients 2 and 3, ripretinib and total concentrations reached C_max_ at around 2 h. And for patient 4, all analytes reached C_max_ at 4.06 h. The t_1/2_ of ripretinib and DP-5439 varied considerably between patients, with a median t_1/2_ (range) of 22.04 (15.58∼26.49) hours for the total concentration at the 150 mg QD dosage, compared to 9.06 h in patient 4 with 150 mg BID. The median area under the concentration-time curve from 0 to 24 h (AUC_0-24h_) were 17,720.43 μg/L·h for ripretinib and 15,664.93 μg/L·h for DP-5439 in patients treated with 150 mg QD. For patient 4, the area under the concentration-time curve from 0 to 12 h (AUC_0-12h_) were 7,055.65 μg/L·h for ripretinib and 12,601.33 μg/L·h for DP-5439.

**TABLE 6:** The main pharmacokinetic parameters of four patients received ripretinib.

Parameter	Patient 1			Patient 2			Patient 3				Patient 4	
	Ripretinib	DP-5439	Total	Ripretinib	DP-5439	Total	Ripretinib	DP-5439	Total	Ripretinib	DP-5439	Total
AUC_0-12h_(μg/L·h)	11,867.56	10,877.68	22,754.81	10,713.73	8,099.59	18,828.60	8,986.78	4,864.35	13,856.58	7,055.65	12,601.33	19,659.58
AUC_0-24h_(μg/L·h)	18,350.24	19,197.39	37,645.14	17,720.43	15,664.93	33,680.33	14,997.88	8,686.42	23,707.87	—	—	—
t_1/2_ (h)	12.18	35.78	22.04	14.18	62.95	26.49	15.58	26.78	18.63	7.94	9.84	9.06
T_max_ (h)	6	0.57	6	2.07	6.07	2.07	2.25	2.25	2.25	4.06	4.06	4.06
C_max_ (μg/L)	1,181.88	989.28	2,160.97	1,287.09	752.30	1934.26	1,072.28	463.70	1,535.98	812.98	1,249.39	2062.37
T_min_ (h)	24	24	24	24	0	24	23.83	23.83	23.83	0.57	0	0
C_min_ (μg/L)	376.59	644.80	1,021.39	320.82	529.89	910.53	355.68	266.63	622.31	332.05	914.31	1,253.34
V_d_ (L)	143.63	403.31	126.69	0.17	0.87	0.17	0.22	0.67	0.17	243.47	168.95	99.73

AUC_0–12_, area under the concentration-time curve from 0 to 12 h posetdose; AUC_0–24h_, area under the concentration-time curve from 0 to 24 h posetdose; t_1/2_, terminal half-life; T_max_, time to maximum plasma concentration; C_max_, maximum plasma concentration; C_min_, trough concentration; V_d_, the volume of distribution at steady state.

## 4 Discussion

This study established and validated an LC-MS/MS method for the determination of ripretinib and its active metabolite DP-5439 in human plasma. The established method was initially implemented for quantifying ripretinib levels in plasma samples from 33 patients who received ripretinib at our hospital and for the pharmacokinetics studies in 4 patients.

To acquire mass spectral data, MRM mode with ESI+ was chosen, consistent with previously published methods for detection in beagles and rats. In the UPLC-MS/MS method developed by Wang et al. (2021) for quantifying ripretinib in beagle dog plasma, the ion transitions were monitored at 509.93→416.85 for ripretinib. While the LC-MS/MS method established by Mudavath and Ashok (2023) in rat plasma employed 510.09→94.06. In the LC-MS/MS method established by Qian et al. (2024) in human plasma, the ion transitions were monitored at 510.1→417.1 for ripretinib and 496.0→403.1 for DP-5439. In this study, ripretinib was monitored with the ion transitions of 510.1→417, and DP-5439 was 496.11→ 402.9, consistent with the results of Qian et al.

In this study, D8-ripretinib, a deuterium substituent analogue of ripretinib, was utilized as the internal standard. This isotopic internal standard closely mirrored the structure of both ripretinib and DP-5439, resulting in a retention time similar to that of ripretinib. This congruence significantly reduced errors arising from matrix interference and disparities in ionization characteristics among analytes. Simultaneously, during LC-MS/MS detection, structurally similar substances can induce ion suppression, potentially compromising detection accuracy. Therefore, we assessed the performance of IS solutions at concentrations of 10.0 μg/mL, 5.0 μg/mL, and 2.0 μg/mL. The results indicated that using an IS concentration of 2.0 μg/L minimized ion suppression, leading to notable improvements in the linearity of the standard curve and significant enhancements in both the accuracy and precision of quality control measures. Furthermore, our method provided a wider linear range (10–5,000 μg/L both for ripretinib and DP-5439) compared to the existing method (Qian et al., 7.5–3,000 μg/L for ripretinib and 10–4,000 ng/mL for DP-5439), thus accommodating the varied demands of clinical sample analysis. The mobile phases used in this method were 0.1% formic acid in water (phase A) and acetonitrile (phase B), similar to that of Wang et al.; however, Qian et al. added 5 mM ammonium formate to mobile phase A to enhance the peak shapes of the analytes. Meanwhile, various chromatographic conditions were explored to improve the specificity and selectivity of ripretinib and DP-5439, including elution methods and gradient elution times. Both gradient elution and isocratic elution (10% A phase: 90% B phase) were assessed. The results indicated that isocratic elution caused peak tailing, while gradient elution facilitated superior separation of distinct analytes. Following evaluations at elution times of 5, 6, and 7 min, it was determined that a 6-min elution time yielded enhanced substance separation and higher response values in a shorter period. Compared to the method of Qian et al. (2024), the analysis time of this method is slightly longer (6 min versus 4.7 min). However, based on the simple preparation of the mobile phase, convenient operation, and lesser internal standard usage, the method of this study made improvements in economic practicability and clinical applications, which may reduce detection costs.

Due to the limited solubility of ripretinib, DP-5439 and D8-ripretinib in both water and methanol, DMSO was used to enhance solubility (Huang et al., 2022). Subsequently, the agents were diluted with methanol-water to prepare the working solution and samples. However, pure methanol solution exhibited high viscosity at room temperature, potentially leading to analyte adsorption onto container walls and resulting in greater inaccuracies. Therefore, to prevent the precipitation of analytes and ensure the stability of samples, it was crucial to prepare the working solution with the highest feasible concentration of methanol. After experimenting with various ratios of methanol-water, it was determined that an 85% methanol-water mixture reduced wall adhesion while maintaining a sufficient concentration of methanol, thereby minimizing solvent effects. For pretreatment, acetonitrile was selected as the protein precipitant in this study for its convenience and cost-effectiveness in therapeutic drug monitoring.

In method validation, we did not assess matrix effects in hyperlipidemic and hemolytic matrices, unlike the study by Qian et al. However, Qian et al. (2024) only reported data from 15 patients receiving 150 mg of ripretinib. In contrast, our study evaluated the method’s applicability on a larger sample size, presenting concentration results for the 150 mg and 300 mg dosage groups. In this study, thorough blood sampling for pharmacokinetic curve analysis was performed on four patients, providing a preliminary description of the pharmacokinetics of ripretinib.

The PK of ripretinib had been preliminarily explored in its phase I clinical study, revealing significant inter-patient variability following ripretinib administration. The geometric mean C_min_ (CV%) reached 284 (62.5%) μg/L for ripretinib and 546 (78.2%) μg/L for DP-5439 after patients attained steady-state levels on a regimen of ripretinib 150 mg QD for more than 15 days. For those who received ripretinib 150 mg BID continuously, the geometric mean C_min_ (CV%) of ripretinib and DP-5439 were 968 (113.8%) μg/L and 1,590 (93.8%) μg/L, respectively (Janku et al., 2020). In this study, the total median C_min_ (range) of patients treated with 150 mg QD for more than 15 consecutive days was 1129.46 (140.95 ∼ 2981.39) μg/L, of which the median C_min_ (range) of ripretinib was 398.50 (66.98 ∼ 1458.91) μg/L, and 654.74 (30.71 ∼ 1522.48) μg/L for DP-5439, slightly surpassing the patient levels in phase I trial (Janku et al., 2020). Xu et al. (2023) conducted a study monitoring the plasma concentration of ripretinib in Chinese patients. Among 42 patients, the C_min_ of ripretinib was 406.55 ± 272.52 (mean ± SD) μg/L, comprising 378.08 ± 226.3 μg/L for 150 mg QD and 863.67 ± 511.7 μg/L for 150 mg BID. Compared to Hao Xu et al.’s study, the C_min_ of ripretinib in this study was marginally elevated. This discrepancy might stem from potential bias attributed to ripretinib high PK parameter variability and the limited patient cohort size in this study.

In the phase I clinical study, the geometric mean C_max_ (CV%) reached 761 (31.8%) μg/L for ripretinib and 804 (45.5%) μg/L for DP-5439 among those receiving 15 consecutive days on a regimen of 150 mg QD (Janku et al., 2020). However, In the Chinese patient cohort, the C_max_ (range) of ripretinib and DP-5439 was observed at 833 (308-1700) μg/L and 1,250 (276-1930) μg/L, respectively (Li et al., 2022a). In this study, C_max_ for all three patients taking 150 mg QD ripretinib was within the range of data from the phase I clinical study and the Chinese patient cohort. Besides, the median AUC_0-12h_ of ripretinib and DP-5439 for 150 mg QD dosage in this study were also consistent with that of the Chinese patient cohort (median AUC_0-12h_(range): ripretinib: 6,610 (2,760–15,900) μg/L·h; DP-5439: 11,400 (2,450–18,600) μg/L·h). For patient 4 with 150 mg BID, C_max_ (ripretinib: 812.98 μg/L, DP-5439: 1,249.39 μg/L) and AUC_0-12h_ (ripretinib: 7,055.65 μg/L·h, DP-5439: 12,601.33 μg/L·h) were slightly below the geometric mean C_max_ (CV%) (ripretinib: 1,290 (79.1%) μg/L, DP-5439: 1,800 (85.9%) μg/L) and AUC_0-12h_ (CV%) (ripretinib: 7,929 (97.7%) μg/L·h, DP-5439: 15,646 (110.3%) μg/L·h) of the Phase I clinical study (Janku et al., 2020).

However, some limitations remain in this study. Due to the small sample size of this study and the short follow-up time, it is unsatisfactory to analyze the correlation based on concentration results and clinical outcomes. In addition, the same high inter-individual variability of PK parameters as in the clinical trial could also be observed in the four patients with intensive blood collection. This also provides a reference for the subsequent pharmacokinetic study of ripretinib and demonstrates new challenges. Further studies are being conducted to expose the correlation between plasma concentrations of ripretinib and clinical benefit in patients with GISTs.

## 5 Conclusion

In conclusion, an LC-MS/MS method capable of concurrently quantifying ripretinib and its metabolite DP-5439 in human plasma was established. Following thorough validation, the method was effectively utilized in PK investigations and TDM of ripretinib among 33 Chinese patients with advanced GISTs. This method provided a swift, straightforward, and precise assay for ripretinib TDM and PK evaluations within clinical settings.

## Data Availability

The raw data supporting the conclusions of this article will be made available by the authors, without undue reservation.
