# Supplementary material for: Liquid chromatography-tandem mass spectrometry for the quantification of ripretinib and its metabolites DP-5439 in human plasma
Source: Front Pharmacol. 2025 Jan 6;15:1506931. doi: 10.3389/fphar.2024.1506931 (PMC11743273; doi:10.3389/fphar.2024.1506931)
Supplement: Supplementary file 1 [file DataSheet1.docx]

Supplementary Material

# Supplementary Figures and Tables

## Supplementary Figures

A**
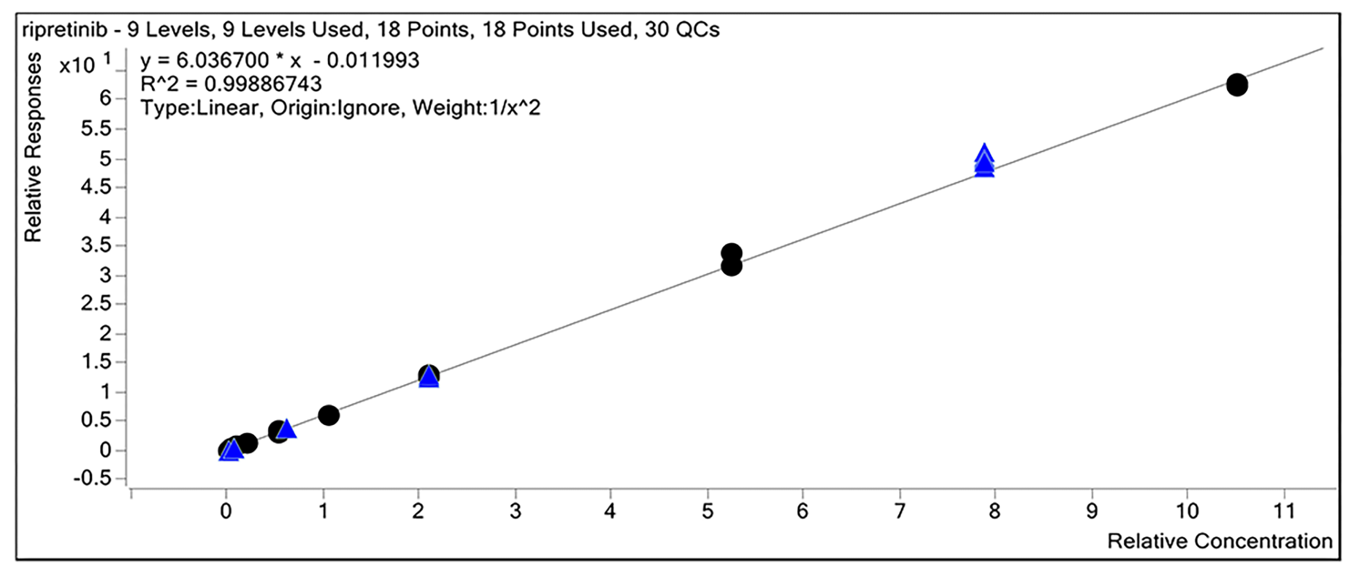
**

B
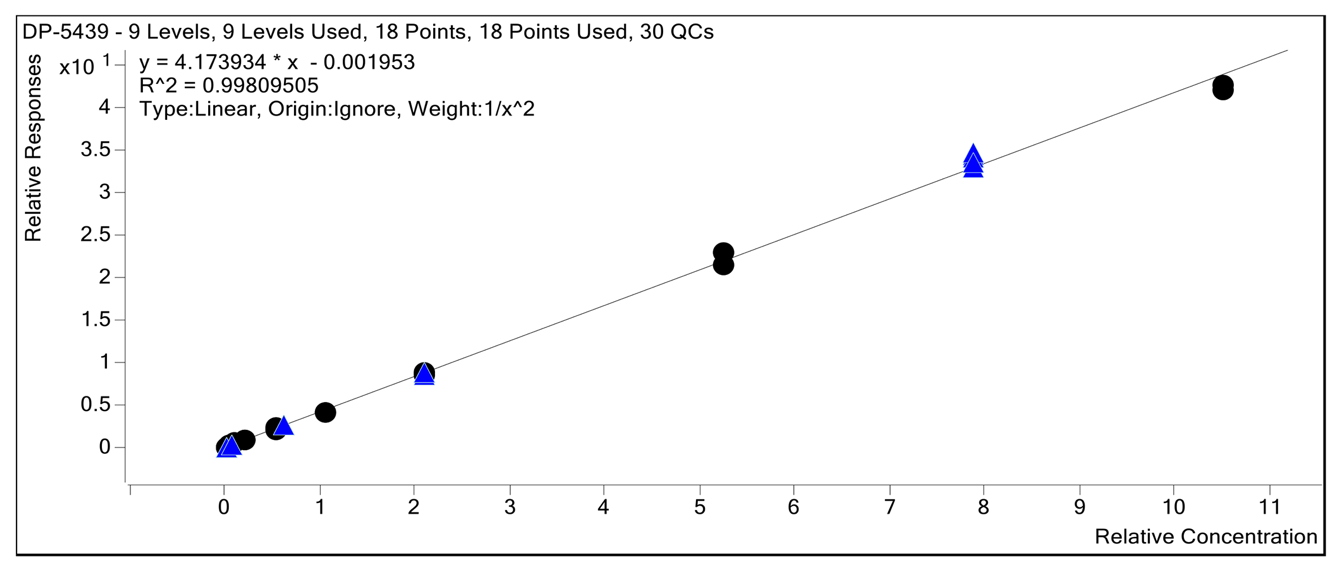
**Supplementary Figure 1.** The linearity graph of ripretinib (A) and DP-5439 (B) in spiked human plasma over the concentration range of 10–5000 μg/L.

## Supplementary tables

Supplementary Table 1. The carry-over effect of ripretinib and DP-5439.

| Analyte | Measurement Level | Nominal Conc.  μg/L | Response | SD | RSD (%) | Analyte response/LLOQ response | Analyte response/IS response |
| --- | --- | --- | --- | --- | --- | --- | --- |
| ripretinib | LLOQ | 10 | 1615.20 | 246.57 | 15.27% |  |  |
|  | Blank | 0 | 232.82 | 27.14 | 11.66% | 14.41% | 1.95% |
| DP-5439 | LLOQ | 10 | 1190.35 | 176.12 | 14.80% |  |  |
|  | Blank | 0 | 212.23 | 24.85 | 11.71% | 17.83% | 1.77% |
| IS | Working standard | 500 | 11961.37 | 3179.52 | 26.58% |  |  |
|  | Blank | 0 | 3.71 | 0.98 | 26.45% | 0.03% | - |

*RSD: Relative standard deviation; SD: Standard deviation, LLOQ, lower limit of quantification. IS, internal standard

Supplementary Table 2. The dilution integrity of ripretinib and DP-5439.

| Analyte | Measurement Level | Nominal Conc.  μg/L | Measured conc. (μg/L) | SD | RSD (%) | RE (%) |
| --- | --- | --- | --- | --- | --- | --- |
| ripretinib | DQC*300 | 25 | 24.07 | 0.95 | 3.95% | -3.73% |
|  | DQC*300 | 250 | 231.92 | 5.83 | 2.51% | -7.23% |
|  | DQC*10 | 750 | 699.68 | 16.96 | 2.42% | -6.71% |
|  | DQC*2 | 3750 | 3541.78 | 104.05 | 2.94% | -5.55% |
| DP-5439 | DQC*300 | 25 | 26.60 | 0.63 | 2.38% | 6.38% |
|  | DQC*300 | 250 | 240.92 | 7.58 | 3.15% | -3.63% |
|  | DQC*10 | 750 | 701.67 | 20.28 | 2.89% | -6.44% |
|  | DQC*2 | 3750 | 3515.07 | 106.46 | 3.03% | -6.26% |

* RSD: Relative standard deviation; RE: Relative error; SD: Standard deviation; DQC: dilution QC, QC, quality control.
